# An Eye-Opening Presentation of Syphilis

**DOI:** 10.7759/cureus.25132

**Published:** 2022-05-19

**Authors:** Natalie Torrente, Jorge Verdecia, Michael Sands

**Affiliations:** 1 Internal Medicine, University of Florida College of Medicine, Jacksonville, USA; 2 Infectious Diseases, University of Florida College of Medicine, Jacksonville, USA

**Keywords:** sexually transmitted infections, neurosyphilis, penicillin, syphilis, anterior uveitis

## Abstract

Syphilis has been called “the great masquerader.” In this case report, we present a case of an HIV-negative patient with left eye redness and decreased vision. Syphilitic anterior uveitis as a complication of neurosyphilis was subsequently diagnosed and the patient showed marked improvement with treatment.

## Introduction

Syphilis is a sexually transmitted disease caused by the spirochete Treponema pallidum [1]. In 2015, the Center for Disease Control and Prevention (CDC) reported a 7.5 per 100,000 population of primary and secondary syphilis. In 2019, there were 129,813 reported new diagnoses of syphilis (all stages). Of these cases, 38,992 were primary and secondary syphilis, the earliest and most transmissible stages of syphilis [2]. Syphilis is seen commonly in men who have sex with men and those with a higher incidence of HIV infection [2,3]. Ocular manifestations of the disease are most commonly seen as posterior uveitis or panuveitis [4]. Given the recent increase in syphilis cases, high clinical suspicion of uveitis as a manifestation of syphilis needs to be present in HIV-negative patients, as syphilitic uveitis can lead to blindness if left untreated. 

This report was published as an abstract at the Southern Medical Association meeting in November 2021.

## Case presentation

**Figure 1:**
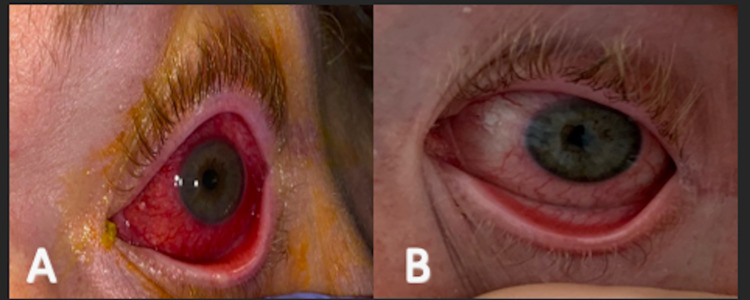
A. Initial presentation with left eye redness. B. Improvement of the left eye after seven days of IV penicillin.

A 43-year-old Caucasian male with a past medical history of non-insulin-dependent diabetes mellitus (last A1c 6.2%) on metformin presented to the emergency room complaining of left eye pain, redness, and decreased vision for three weeks. The onset of his symptoms was gradual, with progressive worsening and associated discomfort to light. The patient denied trauma or an inciting event, itchiness, pain with eye movement, or colored halos. He denied prior fevers, sore throat, cough, or genital or rectal lesions. He was homeless and was living at a shelter facility. He reported that he had been sexually active with multiple partners, males, and females, for the past year. On physical exam, visual acuity was 20/100 in the left eye, the left pupil had a sluggish reaction, accommodation was retained, and the intra-ocular pressure was 21 mmHg. The left eye had diffused conjunctiva injection; the cornea was hazy with punctate epithelial erosions and mild edema. The anterior chamber had inferior keratotic precipitates, cells 1+, flares 3+ and the iris had flat posterior synechiae (Figure 1). There were no skin rashes or lesions present, including on the genital exam. 

**Figure 2:**
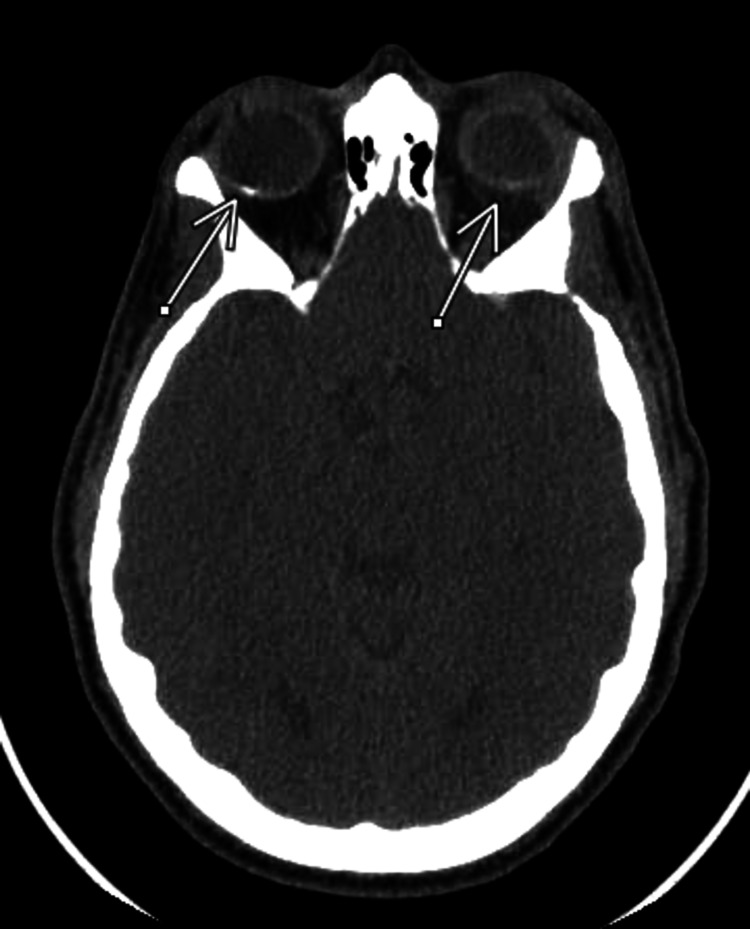
CT of the head showing retinal calcifications (arrows).

On ophthalmology evaluation, there was no evidence of diabetic retinopathy. His HIV antibody-antigen (Ab-Ag) test was negative. He had a positive syphilis serology with a rapid plasma reagin (RPR) of 1:128. CT of the head without contrast showed posterior placoid chorioretinitis (Figure 2). Cerebrospinal fluid (CSF) revealed lymphocytic pleocytosis, WBC 39/UL with 81% lymphocytes, and slightly elevated protein at 54 mg/dL with glucose of 68 mg/dL. Venereal disease research laboratory (VDRL) on the cerebral spinal fluid was reactive. Given his clinical results and the likely diagnosis of syphilitic uveitis, ophthalmology did not proceed with anterior chamber aqueous fluid sampling. 

He was diagnosed with neurosyphilis and associated anterior uveitis and was treated with intravenous penicillin G 24 million units via continuous infusion for 14 days, with rapid eye improvement (Figure 1B). After intravenous therapy, he was given benzathine penicillin G 2.4 million units intramuscular weekly for three weeks. As per ophthalmology recommendations, the patient was given topical atropine TID and pred-forte QID for anterior uveitis. 

## Discussion

Ocular syphilis occurs in about 0.6%-2% of all patients at any stage of the disease. The most common presentations of ocular syphilis are posterior uveitis or panuveitis [4,5]. It occurs more often during the late-latent stage in the immunocompetent elderly population, and may also occur in conjunction with HIV coinfection in younger patients [4,5]. In a study of 143 patients with syphilitic uveitis, patients with isolated anterior uveitis had a 14.5 times higher likelihood of being HIV-positive. They also noted that almost 62% of ocular syphilis in HIV-negative patients presented with posterior uveitis [5]. It has been shown that there is visual loss and worsening visual acuity as well as possible blindness with a longer duration of uveitis; therefore, making a prompt diagnosis and treatment is essential [4].

Acute syphilitic posterior placoid chorioretinitis (ASPPC) was first described in 1990 by Gass et al. as a distinctive ocular manifestation of syphilis [6]. It is characterized by a yellowish, ill-defined, placoid lesion confluent in the posterior pole or mid-periphery of the fundus [7]. The placoid lesions, seen in our case, are an uncommon manifestation typically seen in immunocompromised patients [8]. In a retrospective, multicenter chart review of patients with ASPPC, all patients were treated with the standard treatment for neurosyphilis, IV penicillin G (24 million units/day for 14 days). Follow-up showed that vision improved after antibiotic therapy in both eyes [8]. 

Syphilitic uveitis is equivalent to neurosyphilis, with comparable treatment modalities and duration. The treatment of choice is aqueous crystalline penicillin G 18-24 million units per day, administered as 3-4 million units IV every four hours for 14 days [1]. 

## Conclusions

The patient, in our case report, had a serologic diagnosis of syphilis and had anterior uveitis, which is rare in an immunocompetent patient without HIV infection. In addition, anterior uveitis is one of the minor types of infectious uveitis seen in syphilis patients; studies have shown that posterior uveitis or panuveitis are the most common ocular syphilis manifestations. CSF VDRL and lymphocytic pleocytosis confirmed neurosyphilis diagnosis, and the patient was treated with intravenous penicillin with significant clinical improvement. 
